# Survival Outcomes and Clinicopathologic Prognostic Factors in Gastric Cancer: A Small Single-Center Retrospective Cohort From Eastern Türkiye

**DOI:** 10.7759/cureus.101204

**Published:** 2026-01-09

**Authors:** Sezer Gökçen, Mahmut Baran Yerlikaya, Adem Aslan, Harun Bayram, Tutku Tüfekçi, Yakup Ozan Verendag

**Affiliations:** 1 General Surgery, Ağrı Training and Research Hospital, Ağri, TUR

**Keywords:** gastrectomy, gastric cancer, lymph node ratio, prognostic factors, survival time

## Abstract

Introduction: Real-world data from eastern Türkiye regarding gastric cancer outcomes remain limited. We aimed to describe overall survival (OS) and explore associations between OS and key clinicopathologic factors in a small, single-center cohort.

Methods: We retrospectively reviewed consecutive adults with gastric adenocarcinoma who underwent gastrectomy with curative intent between January 2019 and June 2025. Perioperative mortality (<30 days) was excluded to focus on long-term oncologic outcomes. OS was defined from surgery to death from any cause; survivors were administratively censored at June 2, 2025. Kaplan-Meier methods and univariate Cox models were used.

Results: Thirty patients were included (mean age 65.8±9.4 years; 20/30 male). Median follow-up among survivors was 19.2 months (IQR 8.2-30.8). Twelve- and 24-month OS were 71.1% and 62.2%, respectively; median OS was not reached. Median retrieved lymph nodes was 20 (IQR 19-36; range 4-61). Median lymph node ratio (LNR) was 0.09 (IQR 0.04-0.21). Older age showed a borderline association with worse OS in univariate Cox analysis.

Conclusions: In this small exploratory cohort, OS estimates were modest and associations with clinicopathologic variables were largely inconclusive due to limited power. Larger, multicenter cohorts with standardized reporting of histology, staging, and perioperative treatment are required.

## Introduction

Gastric cancer remains a major contributor to global cancer mortality and continues to pose substantial challenges in resource-limited settings [1]. Contemporary management strategies are guided by evidence-based clinical practice recommendations, particularly regarding multimodal treatment for resectable disease [2]. Histologic subtype (e.g., Lauren intestinal vs diffuse type) reflects tumor biology and may influence clinical behavior [3]. Standard prognostic variables include depth of invasion (pT), nodal status, and composite staging systems [4]. Perioperative chemotherapy has improved survival compared with surgery alone in appropriately selected patients [5,6].

Regional outcome data from eastern Türkiye are relatively sparse. Reporting local experience can help identify practice gaps (e.g., variability in perioperative therapy) and inform quality improvement efforts. Adequate lymphadenectomy and pathologic assessment - including retrieval and evaluation of sufficient lymph nodes - are central to accurate staging and survival estimation in gastric cancer [7,8].

This pilot study aimed to (i) describe overall survival (OS) after curative-intent gastrectomy for gastric adenocarcinoma at a single center in eastern Türkiye, and (ii) explore associations between OS and key clinicopathologic factors, including surgery type, pT stage, lymph node ratio (LNR) [9,10], vascular invasion (lymphovascular invasion (LVI)/perineural invasion (PNI)) [11], margin status [12], and perioperative systemic therapy.

## Materials and methods

Study design and setting

This retrospective cohort study was conducted at a single tertiary hospital in eastern Türkiye.

Eligibility criteria

Adults (≥18 years) with pathologically confirmed gastric adenocarcinoma who underwent gastrectomy with curative intent between January 2019 and June 2025 were eligible. Patients with perioperative mortality (<30 days after surgery) were excluded to focus on long-term oncologic survival and to reduce distortion of OS estimates by early postoperative deaths.

Variables and definitions

Clinicodemographic variables (age, sex), perioperative risk markers (American Society of Anesthesiologists (ASA) class, Charlson Comorbidity Index), nutritional measures (preoperative albumin, BMI), pathology (tumor size and location, Lauren type, grade, pT/pN category, American Joint Committee on Cancer (AJCC) stage when available, LVI, PNI, resection margin status (R0/R1/R2)), and treatment (neoadjuvant chemotherapy, adjuvant chemotherapy; recorded as binary indicators and regimen labels when available) were extracted from electronic medical records.

Outcome definition

OS was defined as time from surgery to death from any cause. Patients without a recorded death were censored at last known follow-up; if last follow-up date was not available in the dataset, an administrative censoring date of June 2, 2025, was applied.

Statistical analysis

Descriptive statistics were reported as mean±SD or median (IQR) for continuous variables and as n (%) for categorical variables. Kaplan-Meier methods were used to estimate OS; between-group comparisons used the log-rank test. Exploratory univariate Cox proportional hazards models were fitted for selected covariates. Given the small sample size and limited number of events, multivariable modeling was not performed.

Ethical considerations

The protocol was approved by the Institutional Ethics Committee of Ağrı İbrahim Çeçen University (Approval No: E-135607; Date: 29.05.2025). Given the retrospective design and use of de-identified data, informed consent was waived.

## Results

A total of 30 patients met the inclusion criteria. The cohort included 20 males (66.7%) with a mean age of 65.8±9.4 years. Baseline clinicopathologic characteristics are summarized in Table 1.

**Table 1:** Clinicopathological characteristics of the study cohort. LVI: lymphovascular invasion, PNI: perineural invasion, ASA: American Society of Anesthesiologists, AJCC: American Joint Committee on Cancer

Characteristic	Value
Age, years	65.8 ± 9.4
Male sex, n (%)	20 (66.7)
ASA class, n (%)	II: 16 (53.3), III: 10 (33.3), IV: 2 (6.7), I: 2 (6.7)
Charlson Comorbidity Index, median (IQR)	3.5 (3.0–4.0)
Preoperative albumin (g/dL), median (IQR)	3.45 (3.22–3.84)
BMI (kg/m²), median (IQR)	23.8 (21.2–27.2)
Surgery type, n (%)	Total 22 (73.3); Subtotal 8 (26.7)
Tumor location, n (%)	Lesser curvature: 11 (36.7), Kardia: 11 (36.7), Antrum: 4 (13.3), Antropyloric: 4 (13.3)
Tumor size (cm), median (IQR); range	3.5 (3.0–4.9); 0.7–8.3
Lauren type, n (%)	Intestinal 12 (40.0); Diffuse 13 (43.3); Mixed 5 (16.7)
pT category, n (%)	pT3: 12 (40.0), pT4a: 10 (33.3), pT1b: 5 (16.7), pT1a: 3 (10.0)
pN category, n (%)	pN1: 13 (43.3), pN2: 7 (23.3), pN0: 4 (13.3), pNx: 4 (13.3), pN3b: 2 (6.7)
AJCC stage (available in 26), n	I 8; II 8; III 10; IV 0
Retrieved lymph nodes, median (IQR); range	20 (19–36); 4–61
Positive lymph nodes, median (IQR); range	2 (1–4); 0–19
Lymph node ratio (LNR), median (IQR)	0.09 (0.04–0.21)
LVI present, n (%)	19 (63.3)
PNI present, n (%)	10 (33.3)
Resection margin, n (%)	R0: 24 (80.0), R1: 4 (13.3), R2: 2 (6.7)
Neoadjuvant chemotherapy, n (%)	3 (10.0)
Adjuvant chemotherapy, n (%)	25 (83.3)
Perioperative systemic therapy (any), n (%)	26 (86.7)

Surgery was total gastrectomy in 22 patients (73.3%) and subtotal gastrectomy in eight (26.7%). Lauren histology was intestinal in 12 (40.0%), diffuse in 13 (43.3%), and mixed in five (16.7%). LVI was present in 19 (63.3%) and PNI in 10 (33.3%). R0 margins were achieved in 24 (80.0%).

AJCC stage was available for 26/30 patients: stage I in eight, stage II in eight, stage III in 10, and stage IV in zero. Perioperative systemic therapy (neoadjuvant and/or adjuvant chemotherapy) was recorded in 26 patients (86.7%); four patients (13.3%) had no recorded perioperative systemic therapy.

**Figure 1:**
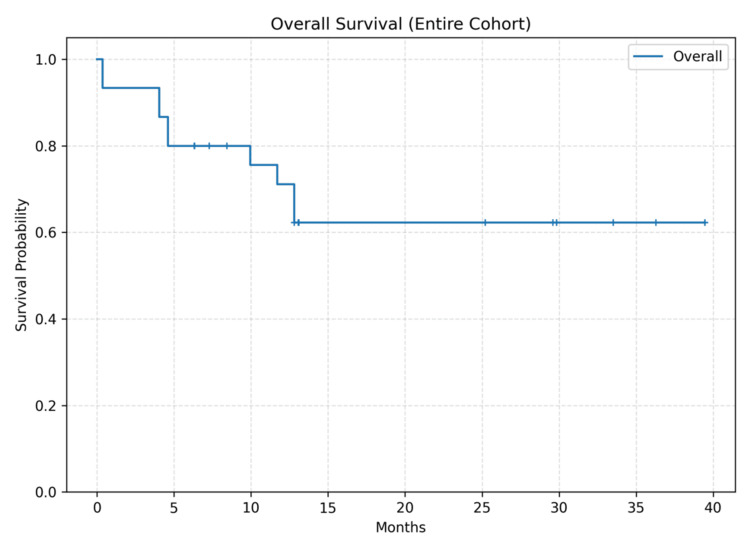
Kaplan-Meier curve for overall survival (OS) in the entire cohort. Kaplan-Meier curve demonstrating overall survival of all patients undergoing curative gastrectomy for gastric adenocarcinoma (n=30). At data censoring (June 2, 2025), 10 deaths had occurred; median OS was not reached. Censored cases are indicated by plus signs.

**Figure 2:**
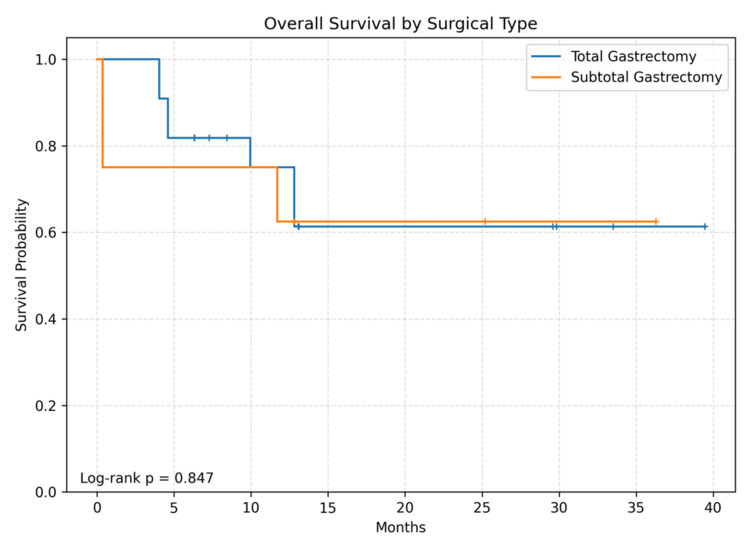
Overall survival according to type of gastrectomy. Kaplan-Meier curves comparing overall survival between subtotal gastrectomy (n=8) and total gastrectomy (n=22). No significant difference was observed (log-rank p=0.847).

**Figure 3:**
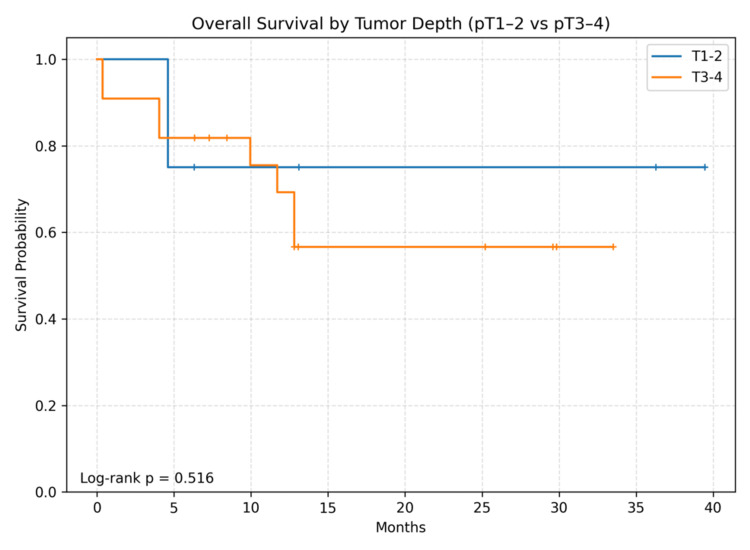
Overall survival according to pathological tumor depth category. Kaplan-Meier curves comparing overall survival between pT1-2 (n=8) and pT3-4 (n=22). No statistically significant difference was observed (log-rank p=0.516).

**Figure 4:**
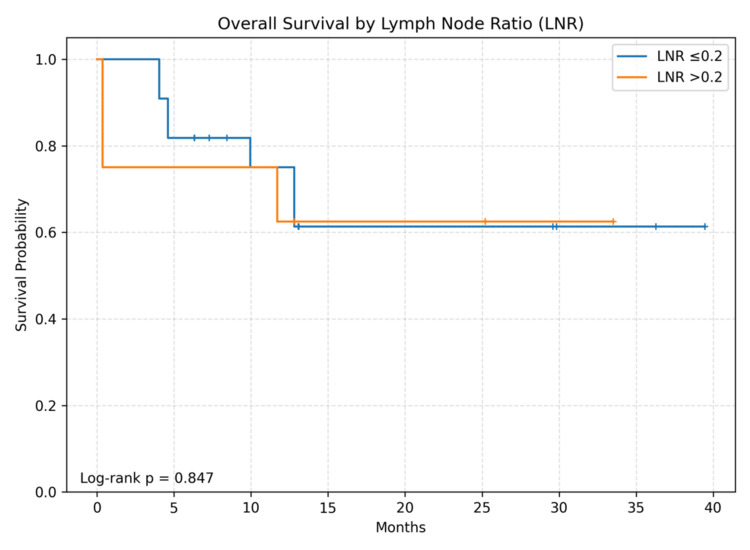
Overall survival according to lymph node ratio (LNR). Kaplan-Meier curves comparing overall survival for patients with LNR ≤0.2 (n=22) and LNR >0.2 (n=8). No significant difference was observed (log-rank p=0.847).

During follow-up, 10 deaths occurred. Median follow-up among survivors was 19.2 months (IQR 8.2-30.8). Twelve- and 24-month OS were 71.1% and 62.2%, respectively; median OS was not reached. The Kaplan-Meier OS curve for the entire cohort is shown in Figure 1. Kaplan-Meier curves stratified by surgery type, pT category, and LNR are shown in Figures 2-4. Between-group OS differences were not statistically significant for surgery type (log-rank p=0.847), pT group (p=0.516), or LNR cut-off 0.2 (p=0.847).

Exploratory univariate Cox analyses are summarized in Table 2. Older age showed a borderline association with worse OS.

**Table 2:** Univariate Cox proportional hazards regression for overall survival. LNR: lymph node ratio

Variable	N	Events	HR	CI	p
Age (per year)	30	10	1.09	1.00-1.20	0.054
Total vs Subtotal	30	10	0.88	0.22-3.42	0.847
pT3–4 vs pT1–2	30	10	1.65	0.35-7.77	0.516
LNR >0.2 vs ≤0.2	30	10	1.14	0.29-4.45	0.847
LVI present	30	10	0.67	0.19-2.40	0.536
R1–2 vs R0	30	10	0.93	0.20-4.37	0.924
Periop systemic therapy (any)	30	10		Not estimable	NA
Diffuse vs Intestinal (Lauren)	25	9	1.34	0.36-5.04	0.662

## Discussion

In this small exploratory single-center cohort from eastern Türkiye, we observed 12- and 24-month OS estimates of approximately 71% and 62%, respectively, with median OS not reached. Consistent with the limited number of events (n=10), survival comparisons across surgery type, pT category, and the pre-specified LNR threshold of 0.2 were not statistically significant. These findings should be interpreted as descriptive rather than confirmatory.

Tumor biology and vascular invasion

A substantial proportion of tumors demonstrated LVI and PNI, both of which are recognized adverse pathologic features. In gastric cancer, LVI has been repeatedly associated with nodal involvement, recurrence, and inferior survival, including in early-stage disease, and is often considered when selecting patients for adjuvant therapy. Given our small sample, we did not observe a statistically robust association between LVI and OS, but the high prevalence of LVI/PNI in this cohort supports the clinical impression of biologically aggressive disease and underscores the importance of systematic pathologic reporting and standardized risk stratification [11].

Histologic subtype and staging

We reported Lauren subtype distribution (intestinal, diffuse, mixed) and AJCC stage when available, addressing a key replicability concern. Diffuse-type tumors are frequently associated with infiltrative growth patterns and poorer prognosis compared with intestinal type; however, our event count is too small to draw reliable inferences. Stage data were unavailable for a small subset due to incomplete nodal assessment (pNx), which further limits stage-stratified inference and highlights an actionable quality improvement target for data capture.

Perioperative systemic therapy

Most patients had recorded perioperative chemotherapy, with adjuvant therapy being much more common than neoadjuvant therapy. In contemporary practice, perioperative regimens (e.g., fluorouracil, leucovorin, oxaliplatin, and docetaxel (FLOT)-based chemotherapy) are widely used for locally advanced resectable gastric cancer and improve survival compared with older regimens [5,6]. In our dataset, a minority of patients had no recorded perioperative systemic therapy. In routine clinical settings, this may reflect early pathologic stage, frailty/comorbidity, postoperative complications, limited access, or patient preference; however, retrospective chart abstraction may also under-capture timing/regimen details. This observation emphasizes that future regional registries should include standardized fields capturing regimen, cycles, and reasons for omission.

Margin status

Achieving an R0 resection is an important surgical quality and prognostic marker. While most patients achieved R0 margins, a clinically meaningful subset had R1/R2 margins. Meta-analytic evidence suggests that microscopic margin positivity (R1) is associated with inferior long-term survival after curative-intent resection, although confounding by tumor biology and stage is substantial [12]. Our study was underpowered to evaluate the independent impact of margin status.

Why exclude 30-day mortality

Excluding perioperative mortality (<30 days) is a defensible approach when the goal is to characterize long-term oncologic survival and to avoid early postoperative deaths overwhelming cancer-related outcome signals. We explicitly report this criterion to enhance transparency and replicability.

Limitations

The main limitation is the small cohort size and low number of events, which restrict statistical power and preclude multivariable adjustment. The retrospective design introduces selection bias and incomplete data capture (e.g., missing stage for pNx cases; limited documentation regarding multidisciplinary tumor board decisions and detailed chemotherapy regimens). Follow-up duration is relatively short for definitive oncologic outcomes, and administrative censoring was applied when follow-up dates were unavailable.

Despite these limitations, this study provides a transparent snapshot of local practice and outcomes and identifies concrete areas for future improvement: standardized pathology reporting (including histology, LVI/PNI, margins), complete staging documentation, and structured recording of perioperative systemic therapy.

## Conclusions

This pilot retrospective cohort suggests modest short-term survival after curative-intent gastrectomy in eastern Türkiye, but associations between OS and clinicopathologic variables were largely inconclusive due to limited power. Future multicenter studies with standardized staging, histology, vascular invasion, margin reporting, and detailed perioperative treatment capture are needed to generate robust, generalizable prognostic estimates.
